# Prevalence of ESBL-producing *Pseudomonas aeruginosa* isolates in Warsaw, Poland, detected by various phenotypic and genotypic methods

**DOI:** 10.1371/journal.pone.0180121

**Published:** 2017-06-28

**Authors:** Agnieszka E. Laudy, Patrycja Róg, Katarzyna Smolińska-Król, Milena Ćmiel, Alicja Słoczyńska, Jan Patzer, Danuta Dzierżanowska, Renata Wolinowska, Bohdan Starościak, Stefan Tyski

**Affiliations:** 1 Department of Pharmaceutical Microbiology, Medical University of Warsaw, Warsaw, Poland; 2 Department of Clinical Microbiology and Immunology, The Children’s Memorial Health Institute, Warsaw, Poland; 3 Department of Antibiotics and Microbiology, National Medicines Institute, Warsaw, Poland; Universidad de Santiago de Compostela, SPAIN

## Abstract

Knowledge of the prevalence of ESBL enzymes among *P*. *aeruginosa* strains compared to the *Enterobacteraiceae* family is limited. The phenotypic tests recommended by EUCAST for the detection of ESBL-producing *Enterobacteriaceae* are not always suited for *P*. *aeruginosa* strains. This is mainly due to the presence of other families of ESBLs in *P*. *aeruginosa* isolates more often than in *Enterobacteriaceae*, production of natural AmpC cephalosporinase and its overexpression, and co-production of metallo-β-lactamases. The aim of this study was to determine the occurrence of ESBLs in *P*. *aeruginosa* isolated from patients from hospitals in Warsaw, to evaluate the ESBL production of these isolates using currently available phenotypic tests, their modifications, multiplex PCR and molecular typing of ESBL-positive isolates by PFGE. Clinical isolates of *P*. *aeruginosa* were collected in 2000-2014 from four Warsaw hospitals. Based on the data obtained in this study, we suggest using three DDST methods with inhibitors, such as clavulanic acid, sulbactam and imipenem, to detect ESBL-producing *P*. *aeruginosa* strains. Depending on the appearance of the plates, we suggest a reduction in the distance between discs with antibiotics to 15 mm and the addition of boronic acid at 0.4 mg per disc. The analysed isolates carried genes encoding ESBL from the families VEB (69 isolates with VEB-9), GES (6 with GES-1, 1 GES-5, 5 GES-13 and 2 with GES-15), OXA-2 (12 with OXA-15, 1 OXA-141, 1 OXA-210, 1 OXA-543 and 1 with OXA-544) and OXA-10 (5 isolates with OXA-74 and one with OXA-142). The most important result of this study was the discovery of three new genes, *bla*_GES-15_, *bla*_OXA-141_ and *bla*_OXA-142_; their nucleotide sequences have been submitted to the NCBI GenBank. It is also very important to note that this is the first report on the epidemiological problem of VEB-9-producing bacterial strains, not only in Poland but also worldwide.

## Introduction

*Pseudomonas aeruginosa* belongs to the group of ESKAPE pathogens (*Enterococcus faecium*, *Staphylococcus aureus*, *Klebsiella pneumoniae*, *Acinetobacter baumannii*, *Pseudomonas aeruginosa* and *Enterobacter* species) that are common causes of life-threatening nosocomial infections among immunocompromised and critically ill patients [1]. Clinical strains of *P*. *aeruginosa* resistant to many classes of antimicrobial agents, including β-lactams, aminoglycosides and fluoroquinolones, are often isolated [2]. However, an increase in strains resistant to the third- and fourth-generation cephalosporins and carbapenems has become a serious clinical problem worldwide. The primary cause of cephalosporin resistance in *P*. *aeruginosa* isolates is the overexpression of the chromosomal AmpC enzyme (mainly resistant to ceftazidime) and the production of the metallo-β-lactamases, MBLs (resistant to cephalosporins and carbapenems) [3,4]. However, ESBL-positive *P*. *aeruginosa* strains that produce extended-spectrum-β-lactamases (ESBLs) are frequently isolated [2,5]. Recently, strains harbouring both *bla*_ESBL_ and *bla*_MBL_ genes were also described [3,6]. At first, the occurrence of ESBL enzymes was associated with *K*. *pneumoniae* strains, which cause nosocomial outbreaks in intensive care units [7]. To date, the different Gram-negative rod species from the *Enterobacteriaceae* family are still the most frequently isolated ESBL-producing strains worldwide [8]. Generally, ESBLs are a group of β-lactamases that hydrolyse penicillins and cephalosporins, including oxyimino-β-lactams (third- and fourth-generation of cephalosporins) and aztreonam, and are inhibited by β-lactamase inhibitors, such as clavulanic acid, sulbactam and tazobactam [7]. The features of these enzymes are utilized in the phenotypic tests used for the detection of ESBL-producing strains. All ESBL-type enzymes are categorized into two structural Ambler classes, A and D. In *P*. *aeruginosa* strains, the ESBL enzymes from both these classes are observed, primarily β-lactamases from the PER, GES [2,9], VEB [2,10], BEL [2,11,12], and PME [13] family (belonging to class A) and from the OXA family (class D), named extended-spectrum class D β-lactamases (ES-OXAs) [2,5,10]. Additionally, in a few *P*. *aeruginosa* isolates, the presence of ESBLs typical to the *Enterobacteriaceae* family, such as TEM, SHV [2,3] and CTX-M-type [2], was described.

It is important that both American (CLSI) [14] and European (EUCAST) [15] recommendations concern the detection of ESBL enzymes by phenotypic tests only in the *Enterobacteriaceae* family. Indeed, by far the most clinically important groups of ESBLs are CTX-M enzymes, followed by the SHV- and TEM-derived ESBLs that are commonly found in *Enterobacteriaceae* [7,16]. In both these international recommendations, the confirmation of ESBL production should be performed using a combination disk test (CDT) or its equivalent, the broth microdilution test [14,15]. Additionally, the EUCAST recommendations allow the use of a double-disk synergy test (DDST) [15]. However, in all these phenotypic methods, only clavulanic acid as the β-lactamase inhibitor is recommended. The clinical strains of *P*. *aeruginosa* produce different families of ESBLs than the *Enterobacteriaceae* strains. Furthermore, some ES-OXAs are less well inhibited by the known β-lactamase inhibitors than ESBLs from class A [5]. In the case of *P*. *aeruginosa*, the presence of inducible chromosomal AmpC β-lactamase or its overexpression is also important. AmpC β-lactamases belonging to class C cephalosporinases contribute to the intrinsic resistance of *P*. *aeruginosa* to penicillin, some cephalosporins (excluding cefepime and ceftazidime) and β-lactams with β-lactamase inhibitors [2]. The level of AmpC production by *P*. *aeruginosa* isolates may interfere with or even hide the detection of ESBLs by phenotypic tests [17,18].

On the other hand, the most microbiological laboratories in the large hospitals, where the infection of *P*. *aeruginosa* ESBL-positive strains occurs, use automated systems for routine susceptibility testing, like for instance Vitek2. These systems do not cover the ESBL detection in non-fermentative Gram-negative rods. The control of the resistance mechanisms detection in standard *P*. *aeruginosa* strains showed that only 12 out of 54 the Spanish laboratories performed the ESBL-detection complementary tests after routine automated susceptibility testing for PER-1 or OXA-161 producing strains, and 8 out of 54 in the case of GES-1-positive strain [19]. Consequently the correct interpretation of β-lactam resistant phenotypes of *P*. *aeruginosa* strains was obtained by only 12, 8 and 2 out of 54 laboratories in the case of strains producing PER-1, OXA-161 or GES-1 enzymes.

The masking effect of the presence of AmpC on the phenotypic detection of ESBLs and the spread of MBL-positive strains are the main causes of no detection of the ESBL-producing *P*. *aeruginosa* isolates. At the same time there is no recommendation to demonstrate the possible presence of ESBL in *P*. *aeruginosa* strains. Additionally, it is considered that there is a lack of phenotypic tests allowing for rapid and easy detection of ES-OXAs. ESBL detection tests for *Enterobacteriaceae* are not suitable for the detection of the all ESBL-type *P*. *aeruginosa* enzymes. This is why so little is known about the prevalence of ESBL enzymes among *P*. *aeruginosa* strains compared to *Enterobacteriaceae* family isolates. Particularly little is known about the occurrence of such strains in Poland. To date, only one outbreak of *P*. *aeruginosa* infection with PER-1 and OXA-74 in a Warsaw hospital has been published [20].

The aim of this study was to determine the occurrence of ESBLs in *P*. *aeruginosa* clinical isolates from patients at Warsaw hospitals and to evaluate the ESBL production of these isolates using currently available phenotypic tests, their modifications, and by multiplex PCR. Moreover, the phenotypic test results were compared with the presence of particular *bla* genes encoding ESBL-type enzymes. Three new nucleotide sequences of ESBLs were discovered and were submitted to GenBank at NCBI.

## Materials and methods

### Bacterial isolates

Nine hundred clinical isolates of *P*. *aeruginosa* obtained from 4 Warsaw hospital microbiological laboratories were examined in this study. These strains were isolated from samples of various clinical materials derived from different patients according to the principle of one isolate from one patient. The isolates were collected in the period 2000-2014 and were initially identified as ceftazidime or/and cefepime resistant *P*. *aeruginosa* strains in microbiological laboratories by routine microbial methods, including API tests (bioMérieux). All strains were stored at -80°C until analysis. Prior to testing, each strain was subcultured twice on TSA (bioMérieux) medium for 24 to 48 h at 30°C to ensure viability.

### Reagents

The following antibiotic disks from Becton Dickinson were used in the study: aztreonam ATM (30 μg), cefepime FEP (30 μg), ceftazidime CAZ-10 (10 μg) and CAZ-30 (30 μg), imipenem IMP (10 μg), amoxicillin with clavulanate AMC (30/10 μg), ampicillin with sulbactam SAM (10/10 μg), piperacillin with tazobactam TZP (30/6 μg), and ceftazidime with clavulanate CAZ-CL (30/10 μg). The disks containing cefepime with clavulanate FEP-CL (30/10 μg) were purchased from BioRad.

During the determination of ESBL production, two different AmpC inhibitors were used: cloxacillin (Sigma) and boronic acid (purchased as phenylboronic acid from Sigma).

### Determination of antibiotic susceptibility

The antibiotic susceptibility of the clinical isolates against ATM, CAZ-10, and FEP was determined on Mueller-Hinton II (MH II) agar plates (Becton Dickinson) using the disc diffusion method, according to the EUCAST guidelines [21]. The assay was validated by susceptibility testing of the reference strain *P*. *aeruginosa* ATCC 27853 against studied antimicrobial agents. The diameters of the growth inhibition zones were interpreted according to EUCAST guidelines [22].

### Phenotypic detection of ESBL production

Detection of the ESBL production by *P*. *aeruginosa* strains was performed on Mueller-Hinton II (MH II) agar plates (Becton Dickinson) with and without cloxacillin (the concentration in the medium was 250 mg/L) [15] using three disc diffusion tests:

1. Double-disc synergy test (DDST) by EUCAST [15] in three modified variants, with a disc containing different ESβL inhibitors: amoxicillin with clavulanate (DDS-AMC test), ampicillin with sulbactam (DDS-SAM test) and piperacillin with tazobactam (DDS-TZP test). In all these DDST variants, discs with ATM, CAZ-30 and FEP were used;
2. Imipenem double-disc synergy test (DDS-IPM) with discs containing CAZ-30, FEP and a disc with IPM as the ESBL inhibitor [18];
3. Two variants of a combination disc test (CDT) that is the extended CLSI confirmatory disc diffusion test using discs with ceftazidime or cefepime alone and discs with these agents plus clavulanate (CAZ/CL test and FEP/CL test, respectively) [15].
  An isolate was recognized as ESBL-positive when a difference of at least 5 mm in the inhibition zones of CAZ-CL versus CAZ-30 or FEP-CL versus FEP was obtained [15].

All DDS tests were interpreted as positive, indicating ESBL-positive strains, when clean extension of the inhibition zone of antibiotics towards the discs with AMC, SAM, TZP or IPM was observed. In the case of an isolate where there were no visible inhibition zones around the discs with β-lactams or the result was inconclusive, boronic acid (0.4 mg per disc) [12] was added to all discs with antibiotics and the DDS tests were carried out again.

### Genotypic detection of ESBL genes

The total DNA from *P*. *aeruginosa* isolates was extracted using a commercial Genomic Mini Kit (A&A Biotechnology). The specific primers used in this study are shown in Table 1. Detection of β-lactamases was carried out in three multiplex PCR reactions: the first detected *bla*_VEB_, *bla*_GES_, *bla*_OXA-10group_ and *bla*_CTX-M2group_ genes and the second detected *bla*_OXA-2group_, *bla*_SHV_ and *bla*_TEM_ genes, while the third detected *bla*_OXA-18_, *bla*_CTX-M-1group_, *bla*_PER_, *bla*_OXA-1group_ and *bla*_BEL_ genes. Amplification was performed using Maxima Hot Start Taq DNA Polymerase (Thermo Scientific). A positive result in the multiplex PCR reaction was confirmed by amplification with a single primer pair. Control strains harbouring *bla*_VEB-1_, *bla*_GES-1_, *bla*_PER-1_, *bla*_BEL-1_, and *bla*_SHV-2a_ (kindly provided by Prof. M.A. Toleman), *bla*_OXA-1_, *bla*_CTX-M2_ (kindly provided by Prof. M. Gniadkowski) and *bla*_OXA-2_ and *bla*_OXA-10_ (isolated in our laboratory and confirmed by sequencing) were used. Moreover, in the isolates carrying the new genes, the presence of class 1 integrons by PCR and location analysis of *bla*_ESBL_ genes within the variable part of integrons were determined.

**Table 1:** Primers used for amplification.

Primer	Target gene(s)	Sequence (5’-3”)	Product size (bp)	Reference
VEB-F	*bla*_VEB group_	CCGATTGCTTTAGCCGTTT	350	this study
VEB-R		GTTCATCGCTGTTGGGGTT		this study
GES-F	*bla*_GES group_	CGCTTCATTCACGCACTATT	517	this study
GES-R		TCTCTGAGGTCGCCAGGT		this study
OXA-1-F	*bla*_OXA-1 group_	TACAGCAGCGCCAGTGCA	710	this study
OXA-1-R		TCGACCCCAAGTTTCCTGT		this study
OXA-2-F	*bla*_OXA-2 group_	TTTTCGATGGGACGGCG	385	this study
OXA-2-R		TCCTACCCACCAACCCAT		this study
OXA-10-F	*bla*_OXA-10 group_	CCGAAGCCGTCAATGGTG	571	this study
OXA-10-R		CCAACCCACCATGCGACA		this study
OXA-18-F	*bla*_OXA-18_	CCATCGTCTGGTATTCGCAG	293	this study
OXA-18-R		CCGGTCTTGCCCTGCAC		this study
PER-F	*bla*_PER group_	GCCTGACGATCTGGAACC	642	this study
PER-R		GCCGTCCATCAGGCAACA		this study
BEL-F	*bla*_BEL group_	GAAACTGCTGCTCTACCCG	788	this study
BEL-R		CGCCTTGCAATTCAGGTGC		this study
SHV-F	*bla*_SHV group_	GCGTTATATTCGCCTGTG	757	this study
SHV-R		GCGCTCTGCTTTGTTATTC		this study
TEM-F	*bla*_TEM group_	GAGTATTCAACATTTCCGTGT	849	[23]^a^
TEM-R		AATCAGTGAGGCACCTATC		[23]^a^
CTX-M1-F	*bla*_CTX-M-1 group_	AAAGTGATGGCCGTGGCC	522	this study
CTX-M2-F	*bla*_CTX-M-2 group_	ATGATGACTCAGAGCATTCGC	749	[24]^a^
CTX-M1+2-R		GATATCGTTGGTGGTGCCA		this study
Int1-F	*intI1*	CCTCCCGCACGATGATC	280	[25]
Int1-R		TCCACGCATCGTCAGGC		[25]
INT-F	variable part of integron	TCGATGTTTGATGTTATGGAGC	variable no. bp	this study
INT-R		AGACTTGACCTGATAGTTTG		this study

F, the forward primer for amplification; R, the reverse primer for amplification.

^a^ starters were slightly modified.

### Sequencing and nucleotide sequence analysis

DNA sequencing was performed at the Laboratory of DNA Sequencing and Oligonucleotides Synthesis, Institute of Biochemistry and Biophysics, Polish Academy of Science, Warsaw, Poland.

For the preparation of DNA templates and sequencing, primers that generated the whole reading frame of the *bla*_ESBL_ gene family were used. DNA and amino acid sequence analyses were carried out using the Vector NTI Advance^TM^ 11 program (Invitrogen). The obtained sequences were compared to sequences deposited in GenBank at NCBI.

### Pulsed-field gel electrophoresis (PFGE)

All isolates producing ESBL detected by phenotype tests (ceftazidime and/or cefepime-resistant isolates) were typed by PFGE according to the Seifert et al. 2005 protocol [26] with modifications, using the *SpeI* enzyme (Thermo Scientific). Electrophoresis was performed by using a CHEF DR II system (BioRad). The migration conditions were as follows: switch angle 120, voltage 6 V/cm, temperature 12°C, and a two-blocks program with a total run time of 26 h (the first-block 1-20 s for 20 h and the second-block 15-40 s for an additional 6 h). The genomic DNA of *Salmonella* serotype Braenderup strain (H9812) was digested with *XbaI* (Thermo Scientific) [27] and the Lambda-DNA Ladder PFG Marker (New England BioLabs) were used as the DNA molecular-weight markers. PFGE patterns were analysed using GelCompar II software (Applied Maths, Belgium) with the Dice coefficient and clustering by UPGMA with 1% tolerance. According to the recommendation of Tenover et al. [28], the isolates were clustered in the PFGE pulsotypes (PTs).

## Results

### Phenotypic detection of ESBL-type enzymes

Among the 900 studied *P*. *aeruginosa* isolates initially described as resistant to third-generation cephalosporins, in the case of 720 isolates (80%), resistance to ceftazidime or/and cefepime was confirmed by the disc diffusion method. In 180 out of 900 (20%) isolates, the inhibition zones around the discs with cephalosporins were similar to the zone diameter breakpoints. ESBL-type enzyme production was detected in 110 out of 720 isolates (15%) in at least one of the phenotypic assays. The largest number of ESBL-positive isolates was found using the DDS-SAM test (92 out of 110 isolates) and then by using CDT with ceftazidime, DDS-AMC and DDS-IMP (77, 76 and 75 isolates, respectively). The information describing the detection of ESBL-positive *P*. *aeruginosa* isolates by different phenotypic tests in relation to the resistance profiles of the studied strains is shown in Table 2. The largest group, 79 out of 110 ESBL-positive isolates, were resistant to three antibiotics, CAZ, FEP and ATM. The second group, 21 out of 110, consisted of isolates that were resistant to CAZ and FEP and at the same time sensitive to ATM. The largest percentage of ESBL-positive strains among these two groups were detected using the DDS-SAM test, 93.7% (74 out of 79) and 71.4% (15 out of 21) isolates. Interestingly, for some of the first group of strains resistant to CAZ, FEP and ATM, it was necessary to conduct tests in the presence of boronic acid, especially in the case of DDS-AMC (31.6%, 18 out of 57 ESBL-positive isolates) and CDT with cefepime (56.3%, 18 out of 32). Generally, for the majority of ESBL-positive isolates, clean extension of the inhibition zone of ceftazidime as well as cefepime towards discs with AMC, SAM, TZP or IPM was observed. However, unlike cefepim, when using discs with ceftazidime in the extended CLSI confirmatory disc diffusion test, a large percentage of ESBL-positive isolates, 82.3% (65 out of 79) in the first group and 52.4% (11 out of 21) in the second group, was observed.

**Table 2:** Detection of ESBL producing *P*. *aeruginosa* isolates by phenotypic tests (in 110 isolates which were resistant to CAZ and/or FEP and/or ATM).

Resistance profiles of isolates	No. of positive isolates											
	DDS-AMC		DDS-SAM		DDS-TZP		DDS-IMP		CAZ/CL		FEP/CL	
	MH	+CX^a^/BA^b^	MH	+CX^a^/BA^b^	MH	+CX^a^/BA^b^	MH	+CX^a^/BA^b^	MH	+CX^a^/BA^b^	MH	+CX^a^/BA^b^
CAZ, FEP, ATM (n = 79)	37	2/18	67	2/5	13	0/6	42	6/6	60	0/5	14	0/18
CAZ, FEP (n = 21)	12	0/0	15	0/0	5	0/0	13	1/2	11	0/0	3	0/0
FEP, ATM (n = 1)	0	0/0	0	0/0	0	0/0	1	0	0	0/0	0	0/0
CAZ (n = 6)	3	1/0	3	0/0	0	0/0	3	0/0	1	0/0	0	0/0
FEP (n = 3)	1	2/0	0	0/0	0	0/0	1	0/0	0	0/0	1	0/0
Total (n = 110)	53	5/18	85	2/5	18	0/6	60	7/8	72	0/5	18	0/18

DDS, double-disc synergy test with different ESBL inhibitors; AMC, amoxicillin with clavulanate; SAM, ampicillin with sulbactam; TZP, piperacillin with tazobactam; IMP, imipenem; CAZ/CL, Combination disc test with ceftazidime and clavulanate; FEP/CL, combination disc test with cefepime and clavulanate; CAZ, ceftazidime; FEP, cefepime; ATM, aztreonam; MH, Mueller-Hinton II medium; +CX, MH medium supplemented with cloxacillin (250 mg/L); +BA, On all discs with antibiotics boronic acid (0.4 mg per disc) was added.

^a^ the number of isolates for which a positive result of ESBL-phenotypic test was obtained only when the MH medium was supplemented with cloxacillin.

^b^ the number of isolates for which a negative result of ESBL-phenotypic test was obtained on the MH medium without and with cloxacillin. When boronic acid was added to the discs with antibiotics a positive result was achieved.

### Occurrence of *bla* genes encoding ESBL-type enzymes

Among 110 phenotypically ESBL-positive isolates, 5 groups of *bla* genes encoding ESBL-type enzymes (VEB, GES, OXA-2, OXA-10 and TEM) were detected. Sequence analysis of all PCR *bla* genes detected in multiplex allowed the definition of the produced enzymes (Table 3). The most important observation is that four *P*. *aeruginosa* isolates carried the novel *bla* genes. In 2 out of these 4 isolates, a novel *bla*_GES_ variant close to the *bla*_GES-5_ gene was identified and submitted as *bla*_GES-15_. The new GES-15 had one substitution, Pro167Ser, compared to GES-5, and two amino acid differences, Pro167Ser and Gly170Ser, compared to GES-1. Furthermore, the isolate carried a novel *bla*_OXA-2_ variant submitted as a *bla*_OXA-141_ gene. The new OXA-141 displayed 1 amino acid change at position 162, Gly162Ser. Additionally, sequence analysis detected a third new gene submitted as *bla*_OXA-142_ in one isolate. The new OXA-142 belongs to the OXA-10 ESBL-type family and has two substitutions in the amino acid sequence, Asn76Ser and Gly167Asp. The nucleotide sequences of the new *bla*_GES-15_, *bla*_OXA-141_ and *bla*_OXA-142_ genes were submitted to the GenBank database (accession number GU208678, EF552405 and EU358785, respectively). Moreover, all the new *bla* genes were located within class 1 integrons. All clinical isolates of *P*. *aeruginosa* carrying the new *bla*_GES-15_, *bla*_OXA-141_ and *bla*_OXA-142_ genes showed typical results for ESBL-positive strains in the phenotypic tests.

**Table 3:** The occurrence of *bla* genes encoding ESBL-type of enzymes with regard to the resistance profiles of analysed *P*. *aeruginosa* isolates.

Resistance profiles of isolates	No. isolates carrying family of enzymes (sequencing results)						
	VEB+OXA-10+GES	VEB+OXA-10	VEB	GES	OXA-10	OXA-2	TEM
CAZ, FEP, ATM (n = 79)	5 (VEB-9, OXA-10, GES-13)	54 (VEB-9, OXA-10)	10 (VEB-9)	0	4 (OXA-74)	1 (OXA-141)	0
					1 (OXA-142)		
CAZ, FEP (n = 21)	0	0	0	6 (GES-1)	0	12 (OXA-15)	0
				2 (GES-15)			
FEP, ATM (n = 1)	0	0	0	0	1 (OXA-74)	0	0
CAZ (n = 6)	0	0	0	1 (GES-5)	0	1 (OXA-210)	0
						1 (OXA-543)	
						1 (OXA-544)	
FEP (n = 3)							3 (TEM-1)^a^
Total (n = 110)	5	54	10	9	6	16	3

CAZ, ceftazidime; FEP, cefepime; ATM, aztreonam.

^a^ in three isolates the *bla*_TEM-1*f*_ gene was detected. *bla*_TEM-1*f*_ is one of gene alleles encoding TEM-1 penicillinase and is a precursor to the ESBL genes.

The occurrence of all detected *bla* genes encoding ESBL-type enzymes with regard to the resistance profiles of *P*. *aeruginosa* isolates is presented in Table 3. Among 110 ESBL-positive isolates in phenotypic tests, the presence of *bla* genes encoding ESBL was shown in 99 isolates, including *bla*_VEB-9_ alone or with *bla*_OXA-10,_ as well as with *bla*_GES-13_ in 69 isolates, *bla*_GES_ (*bla*_GES-1_, *bla*_GES-15_) in 8, *bla*_OXA-2_-type (*bla*_OXA-15_, *bla*_OXA-141_, *bla*_OXA-210_, *bla*_OXA-543_ and *bla*_OXA-544_) in 16 and *bla*_OXA-10_-type (*bla*_OXA-74_ and *bla*_OXA-142_) in 6 isolates. Moreover, in one isolate, the *bla*_GES-5_ gene encoding carbapenemase was detected. On the other hand, the sequencing analysis showed the presence of only the *bla*_TEM-1*f*_ gene encoding TEM-1 β-lactamase in three isolates. In the remaining seven ESBL-positive isolates, the genes encoding enzymes of the families VEB, GES, PER, OXA, TEM, SHV, BEL and CTX-M were not detected.

### Comparison of phenotypic tests results with the presence of *bla* genes encoding ESBL-type enzymes

In Table 4, the comparison of the results obtained from phenotypic tests and multiplex PCR detection is presented. In the case of the presence of the *bla*_VEB-9_ gene in the genomes of 69 studied isolates, the DDS-SAM test allowed for the detection of 100% of such ESBL-positive isolates. However, for one out of 10 isolates carrying only the *bla*_VEB-9_ gene, this test was positive only when the medium was supplemented with cloxacillin. Furthermore, it was necessary to use boronic acid in 3 out of 5 isolates producing additional GES-13 and OXA-10, and in 2 out of 54 isolates when only VEB-9 and OXA-10 were produced. Interestingly, the DDS-IMP test was the only phenotypic method allowing the detection of isolates producing GES-1, GES-5, OXA-74 and OXA-142, whereas the DDS-AMC test identified the strains carrying *bla*_OXA-543._ Unlike the DDS tests, the two variants of a combination disk test enabled the identification of only 78% (78 out of 100 isolates) of ESBL-producing isolates, and only 35% of CDT with cefepime (35 out of 100).

**Table 4:** Comparison of phenotypic test results with the presence of *bla* genes encoding ESBL-type enzymes.

Profiles of enzymes (No. isolates)	Total no. of the ESBL-positive isolates detected by phenotypic tests (on MH without cloxacillin / only on MH with cloxacillin / only by using boronic acid)^a^				
	DDS-AMC	DDS-SAM	DDS-IMP	CAZ/CL	FEP/CL
VEB-9 + OXA-10 + GES-13 (n = 5)	5 (0/0/5)	5 (2/0/3)	2 (0/0/2)	5 (0/0/5)	4 (0/0/4)
VEB-9 + OXA-10 (n = 54)	41 (27/1/13)	54 (52/0/2)	35 (29/2/4)	51 (51/0/0)	10 (3/0/7)
VEB-9 (n = 10)	10 (9/1/nt)	10 (9/1/nt)	10 (8/2/nt)	9 (9/0/0)	8 (8/0/0)
GES-1 (n = 6)	0	0	6 (3/1/2)	0	0
GES-5^b^ (n = 1)	0	0	1 (1/nt/nt)	0	0
GES-15 (n = 2)	2 (2/nt/nt)	2 (2/nt/nt)	2 (2/nt/nt)	2 (2/nt/nt)	2 (2/nt/nt)
OXA-74^c^ (n = 5)	0	0	5 (4/1/nt)	0	0
OXA-142^c^ (n = 1)	0	0	1 (1/nt/nt)	0	0
OXA-543^d^ (n = 1)	1 (0/1/nt)	0	0	0	0
OXA-544^d^ (n = 1)	1 (1/nt/nt)	1 (1/nt/nt)	0	1 (1/nt/nt)	0
OXA-15^d^ (n = 12)	10 (10/0/0)	12 (12/nt/nt)	8 (8/0/0)	9 (9/0/0)	1 (1/0/0)
OXA-141^d^ (n = 1)	0	1 (0/1/nt)	1 (0/1/nt)	0	0
OXA-210^d^ (n = 1)	1 (1/nt/nt)	1 (1/nt/nt)	0	0	0
Total (n = 100)	71 (50/3/18)	86 (79/2/5)	71 (56/7/8)	77 (72/0/5)	25 (14/0/11)

MH, Mueller-Hinton II medium; DDS, double-disc synergy test with different ESBL inhibitors; AMC, amoxicillin with clavulanate; SAM, ampicillin with sulbactam; TZP, piperacillin with tazobactam; IMP, imipenem; CAZ/CL, combination disc test with ceftazidime and clavulanate; FEP/CL, combination disc test with cefepime and clavulanate; nt, not tested because earlier for all groups of isolates positive results for tests on MH medium without cloxacilli / or on MH supplemented with cloxacillin were obtained.

^a^ in parentheses, the number of isolates for which a positive result of ESBL-phenotypic test was obtained on MH medium without cloxacillin / only on agar plates supplemented cloxacillin / only in the presence of boronic acid. The tests with the discs containing antibiotics and also boronic acid were performed only in the case of negative results on the medium without and with cloxacillin.

^b^ enzyme with carbapenemase activity.

^c^ enzymes belong to the OXA-10 family.

^d^ enzymes belong to the OXA-2 family.

Finally, the use of all three DDS tests (DDS-AMC, DDS-SAM and DDSA-IMP) allowed for phenotypic detection of 100% of studied isolates that produced ESBLs (Table 4). However, the detection of single isolates producing VEB-9, GES-1, OXA-74, OXA-543 or OXA-141 enzymes was possible due to the determination of ESBL-production by three DDS tests on the medium with cloxacillin. Carrying out these DDS tests in the presence of boronic acid, the next nine ESBL-positive isolates (two isolates carrying *bla*_GES-1_, two other with *bla*_VEB-9_ and *bla*_OXA-10_, and also three carrying *bla*_GES-13_, *bla*_VEB-9_ and *bla*_OXA-10_) were detected.

### Molecular typing of ESBL-positive isolates

**Fig 1:**
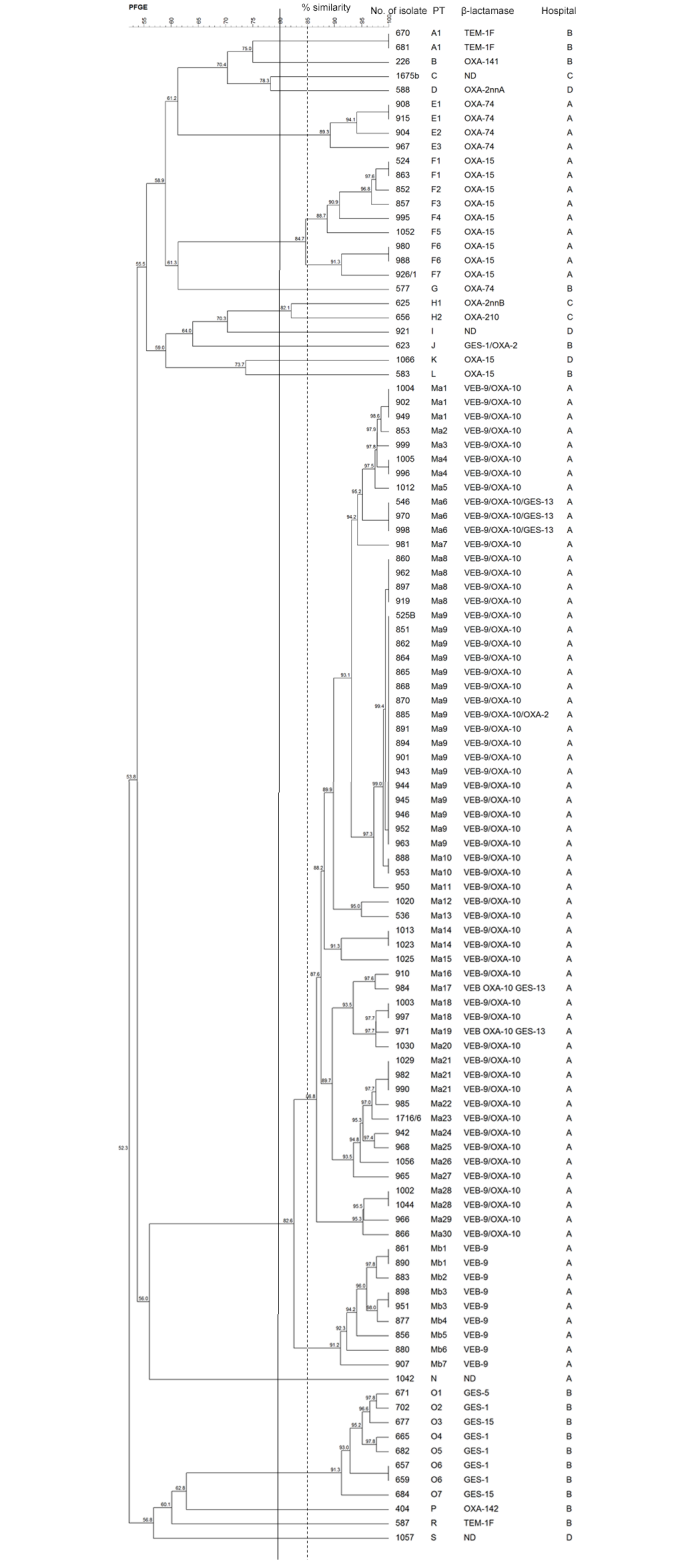
Analysis of PFGE patterns. Dendrogram presents PFGE profiles similarity, the presence of β-lactamases and the origin of *P*. *aeruginosa* clinical isolates that revealed production of ESBL-type enzymes in phenotypic tests. The solid line indicates 80% similarity and was used to define PFGE type. The dotted line indicates 85% similarity and marks its division into the PT subclusters. ND, genes encoding β-lactamases from studied ESBL families were not detected by multiplex PCR.

All 110 ESBL-positive clinical isolates of *P*. *aeruginosa* as determined in phenotypic tests were genotyped by PFGE. Twenty-two different pulsotypes were obtained, from which 18 with a similarity of over 50% are presented in Fig 1. Isolates were considered as a cluster if the similarity was over 80%. The PFGE molecular typing showed the relatedness between all 69 VEB-9-positive *P*. *aeruginosa* isolates obtained from different patients from one Warsaw hospital (Hospital A) during the years 2010-2014. Among these isolates belonging to PT M, two subclusters with greater than 85% similarity could be distinguished, PT Ma (isolates carried the *bla*_VEB-9_ and *bla*_OXA-10_ genes and these isolates additionally with the *bla*_GES-13_ gene) and PT Mb (carried only the *bla*_VEB-9_ gene).

## Discussion

A wide panel of phenotypic methods applied in this study allowed for the detection of *P*. *aeruginosa* isolates producing various ESBL-type enzymes. Clinical isolates from patients from Warsaw hospitals carried genes encoding ESBL from the following families: VEB (69 isolates with VEB-9), GES (6 with GES-1, 5 GES-13 and 2 with GES-15), OXA-2 (12 with OXA-15, 1 OXA-141, 1 OXA-210, 1 OXA-543 and 1 with OXA-544) and OXA-10 (5 isolates with OXA-74 and one with OXA-142). The most important achievement of this study was the discovery of three new genes, named *bla*_GES-15_, *bla*_OXA-141_ and *bla*_OXA-142_, whose nucleotide sequences have been submitted to GenBank (accession number GU208678, EF552405 and EU358785, respectively). Among *P*. *aeruginosa* strains, the OXAs were the most common and the most frequently isolated enzymes worldwide. Besides most of the ES-OXAs have been identified in *P*. *aeruginosa* strains [2]. Therefore, it is not surprising that the same ES-OXAs were found at the same time in a different part of the world. Outside Poland, the presence of OXA-142 was also detected in *P*. *aeruginosa* strains isolated in Bulgaria in 2008-2009 (3 isolates) [10] and Korea (in 4 isolates) [3]. In the case of the Polish strain, the variable part of the class 1 integron contained the gene cassette array *aac(6)-Ib-bla*_OXA-142_*–aadA6/aadA10* (NCBI GenBank acc. no EU358785). Four isolates producing OXA-142 in Korea had a class 1 integron with the order of gene cassettes (*aacA4- bla*_OXA-142_*–aadA2)* identical to the previously submitted sequence of a plasmid-located class 1 integron from another Korean *P*. *aeruginosa* strain (NCBI GenBank acc. no KF257849). Our studies are the first to describe an OXA-141-producing bacteria. To date, in only one *P*. *aeruginosa* strain, isolated in the year 2000, the *bla*_OXA-141_ gene encoding a new ESBL enzyme was observed. Conting 67 of the whole genome shotgun sequence of *P*. *aeruginosa* strain WH-SGI-V-07247, which also contains this gene (accession no. LLTH01000109), has been recently submitted to GenBank. Our work is also the first in which two additional new ESBL-type enzymes from the OXA-2 family, OXA-543 and OXA-544, were found in *P*. *aeruginosa* clinical strains. Unfortunately, only the nucleotide sequences of contings from whole genome shotgun sequences of *P*. *aeruginosa* strains carrying these genes were submitted to GenBank, without specifying the range of activity of these new enzymes (acc. no LLTZ01000057 and LLMY01000078, respectively). Our research showed that *P*. *aeruginosa* isolates carrying these two new enzymes, OXA-543 and OXA-544, have demonstrated typical results for ESBL-positive strains in phenotypic tests. Moreover, among the studied isolates, the presence of genes encoding the OXA-15, OXA-74 and OXA-210 enzymes was demonstrated. The OXA-15 β-lactamase was identified inside a class 1 integron detected in *P*. *aeruginosa* strain from Turkey [29]. The presence of OXA-74 in the *P*. *aeruginosa* clinical isolates was described for the first time in 2007 in Poland [20], and then only in 2008 in Hungary (2 isolates) [30] and in 2013 in Bulgaria (2 isolates) [10], whereas the presence of the gene encoding OXA-210 was demonstrated so far in only one *P*. *aeruginosa* strain isolated in Korea [3]. So, this research is the second reported case of isolating an OXA-210-producing strain in another part of the world—Poland.

An equally important observation of this study is the first report of the epidemiological problem with VEB-9-producing bacterial strains not only in Poland but also worldwide. The PFGE molecular typing showed the relatedness between all 69 VEB-9-positive *P*. *aeruginosa* isolates obtained from different patients from one Warsaw hospital during 2010-2014. Only 5 of 59 isolates grouped in the subcluster PT Ma with greater than 85% similarity also carried the *bla*_GES-13_ gene. Perhaps there has been an acquisition of the *bla*_GES-13_ gene by strains originally possessing only *bla*_VEB-9_ and *bla*_OXA-10_ genes. At first, the VEB-9 enzyme was observed in two *P*. *aeruginosa* isolates from Kuwait in 2001 [31]. To date, there are no other publications reporting the detection of VEB-9-producing strains worldwide. Recently, the whole genome shotgun sequences of three strains, *P*. *aeruginosa* AR_0092 scaffold00001 (acc. no. MPBS01000001), *Acinetobacter baumannii* ABBL 142 contig-1000047 (acc. no LLIK01000044) and *K*. *pneumoniae* OC511 AOT23_contig000137 (acc. no LOEI01000136), which also contain the *bla*_VEB-9_ gene, were submitted to GenBank. Among the *P*. *aeruginosa* clinical worldwide isolates, the VEB-1 enzyme was detected the most frequently [2,6,10,32].

Other important data obtained in this research include a large variety of enzymes from the GES family (GES-1, -5, -13 and GES-15) detected in *P*. *aeruginosa* isolates from such a small area as the city of Warsaw. Unlike the other detected GES-type enzymes, GES-5 possesses carbapenemase activity [33,34]. GES-1 is the most common of the ESBL-type enzymes of the GES group produced by *P*. *aeruginosa* [2,9] as well as by *Enterobacteriaceae* strains [16] in the world. The presence of GES-13-producing *P*. *aeruginosa* strains was described only twice, for the first time in the case of a MDR strain isolated in 2008 in Greece [35] and the second time in a *P*. *aeruginosa* strain isolated in Canada in 2012 [36]. In Polish isolates, the presence of *bla*_GES-13_ as well as *bla*_VEB-9_ and *bla*_OXA-10_ genes in the same isolate is unusual. This work is the first report of GES-15-producing bacteria in the world. The *bla*_GES-15_ gene was detected in one *P*. *aeruginosa* strain within the variable part of class 1 integron located in the plasmid.

The detection of the various ESBL-type of enzymes in *P*. *aeruginosa* isolates from patients in Warsaw hospitals was possible due to the use of a wide panel of phenotypic methods. We modified and extended a double-disk synergy test, mainly by the incorporation of other clavulanic acid β-lactamase inhibitors, such as sulbactam, tazobactam and imipenem. The ESBL-type enzymes produced by *P*. *aeruginosa* strains are not always inhibited by clavulanic acid [5,18]. The most difficult was to detect the isolates harbouring the genes encoding the GES-type [18,36] or OXA-type [5] of ESBLs. As previously described, synergy between imipenem and ceftazidime was observed in the case of GES-1-producing *P*. *aeruginosa* strains in the disc-diffusion method [18]. Among ES-OXAs, for example, OXA-19 can be inhibited by imipenem [5]. In addition, tazobactam was a more potent inhibitor of GES-type ESBLs (GES-1, GES-13) than other inhibitors, such as clavulanic acid, imipenem and sulbactam [34,35]. Sulbactam was only sporadically used in the CDT test to detect the ESBL-positive co-produced AmpC strains [37]. According to the EUCAST recommendations [15], for *Enterobacteriaceae* with inducible chromosomal AmpC, for the phenotypic detection of ESBL in this work, cefepime and the agar plates containing cloxacillin were included. It described that cefepime is less rapidly inactivated by AmpC cephalosporinases than by ESBLs [38] or even that it is stable to AmpC hydrolysis [15]. Performing the DDST on agar supplemented with cloxacillin strengthened the ability of this test to detect bacterial strains carrying genes encoding ESBLs among *P*. *aeruginosa* isolates [17,23,38]. The greatest effect improving the detection of the ESBL-positive isolates in this work was the reduction in the distance between the discs in DDS tests. In the case of *P*. *aeruginosa* isolates for which the diameter zones of inhibition around the discs was not observed or was very small, the distance between the discs in DDST was reduced from 20 to 15 mm. Generally, performing conventional DDST and the above-described modifications, in 105 of the 110 ESBL-positive *P*. *aeruginosa* strains, the ability to produce these types of enzymes was observed. However, it was necessary to reduce the distance between discs with antibiotics to 15 mm in the case of 56 out of 105 ESBL-positive isolates. However, the phenotypic detection of two isolates carrying the *bla*_GES-1_ gene, two isolates carrying *bla*_VEB-9_ and *bla*_OXA-10_ genes and three isolates carrying *bla*_GES-13_, *bla*_VEB-9_ and *bla*_OXA-10_ genes required not only a reduction in the distance between discs but also the use of boronic acid. Previously, the DDST carried out with the addition of 0.4 mg of boronic acid per disc allowed the detection of BEL-1-positive *P*. *aeruginosa* strains in Belgian hospitals [12]. The data obtained in this study showed that boronic acid has a greater ability than cloxacillin to inhibit cephalosporinase overproduction.

Finally, comparing the occurrence of *bla* genes encoding ESBLs in relation to the results obtained in this research concerning ESBL detection by phenotypic tests, it is concluded that in the case of *P*. *aeruginosa*, modified DDS tests should be used instead of tests dedicated for *Enterobacteriaceae* strains. Based on the data obtained in this study, we propose to use three methods (DDS-AMC, DDS-SAM and DDS-IMP) to detect ESBL-producing *P*. *aeruginosa* strains. Depending on the images of the plates, we suggest a reduction in the distance between discs with antibiotics to 15 mm and the addition of boronic acid to the discs. This study is also a good example to demonstrate the effectiveness of the proposed methods in the detection of the various ESBLs, including enzymes from the VEB, GES, OXA-2 and OXA-10 families, which allowed us to determine the occurrence of ESBLs in *P*. *aeruginosa* isolates from clinical materials from patients in Warsaw hospitals.
